# Supplementary figures and images for: Redefining innate natural antibodies as important contributors to anti-tumor immunity
Source: eLife. 2021 Oct 5;10:e69713. doi: 10.7554/eLife.69713 (PMC8547949; doi:10.7554/eLife.69713)

Source data for WB in Figure 4

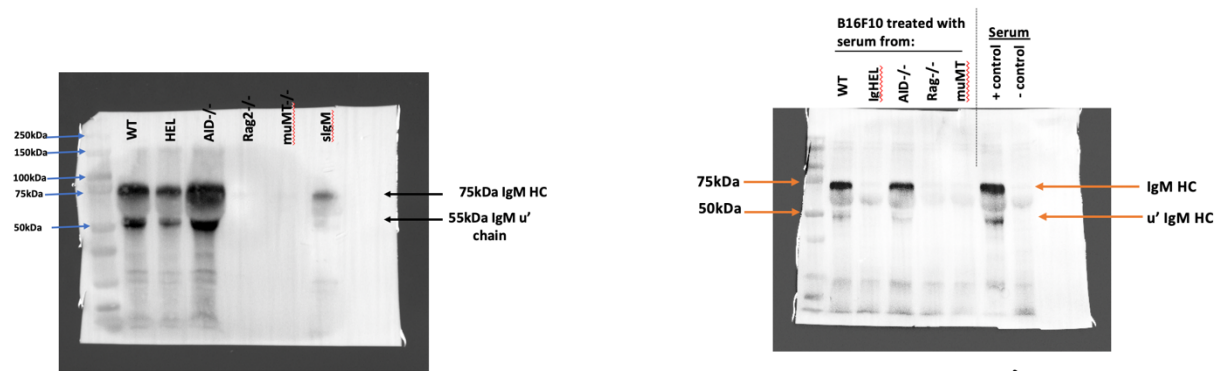

Supplement: Figure 4—source data 1. [file elife-69713-fig4-data1.pdf]
