# Supplementary material for: Redefining innate natural antibodies as important contributors to anti-tumor immunity
Source: eLife. 2021 Oct 5;10:e69713. doi: 10.7554/eLife.69713 (PMC8547949; doi:10.7554/eLife.69713)
Supplement: Supplementary file 1. — *As of597 February 2021. [file elife-69713-supp1.docx]

**Supplementary File 1**

**Table S1.** Articles not supporting and supporting the hypothesis that B cells are essential for early cancer cell recognition and anti-tumor immunity. *As of February 2021

| **Not supporting** | **Title** | **Author (ref)** | **Journal** | **Year** | **Mouse model** | **Cancer type** | ***No. of Citations** |
| --- | --- | --- | --- | --- | --- | --- | --- |
| 1 | B cells inhibit induction of T cell-dependent tumor immunity | Qin et al. (1) | Nature medicine | 1998 | muMT | TS/A cells, J558L cells | 427 |
| 2 | Combination Chemotherapy and IL-15 Administration Induce Permanent Tumor Regression in a Mouse Lung Tumor Model: NK and T Cell-Mediated Effects Antagonized by B Cells | Chapoval et al. (2) | J Immunol | 1998 | muMT | 76-9 cells (syngeneic B6 3-methylcholanthrene-induced) | 98 |
| 3 | Enhanced efficacy of melanoma vaccines in the absence of B lymphocytes | Perricone et al. (3) | J Immunother | 2004 | muMT | B16F10 | 81 |
| 4 | Increased rejection of primary tumors in mice lacking B cells: Inhibition of anti‐tumor CTL and TH1 cytokine responses by B cells | Shah et al. (4) | Tumor Immunology | 2005 | muMT | EL4 thymoma, B16F10 and MC38 colon carcinoma | 202 |
| 5 | Inhibitory Effects of B Cells on Antitumor Immunity | Inoue et. al.  (5) | Cancer Res | 2006 | muMT | Friend murine leukemia virus gag-expressing mouse EL-4, D5 mouse melanoma, or MCA304 mouse sarcoma cells. | 302 |
| 6 | The absence of B lymphocytes reduces the number and function of T-regulatory cells and enhances the anti-tumor response in a murine tumor model | Tadmor et al. (6) | Cancer Immunol Immunother | 2011 | muMT | EMT-6 mammary adenocarcinoma cells | 106 |
| 7 | Immune-promoted tumor cell invasion and Metastasis New consideration in Cancer Therapy | Guillem and Sampsel (7) | Cancer therapy (Google books) | 2012 | muMT | B16F10 and LLC | 4 |
| 8 | B lymphocyte inhibition of anti-tumor response depends on expansion of Treg but is independent of B-cell IL-10 secretion | Zhang et al. (8) | Cancer Immunol Immunother | 2013 | muMT | EMT-6 mammary tumors | 71 |
| 9 | Allogeneic cancer cell-based immunotherapy | Eckhard R Podack (9) | (US PATENT US8475785B2) | 2013 | muMT | EG7, EL4 and LLC cell line | 7 |
| 10 | B cells promote tumor progression in a mouse model of HPV-mediated cervical cancer | Tang et al. (10) | Int. J. Cancer | 2016 | muMT | TC‐1 tumor cells and B16 Ova | 29 |
| 11 | B lymphocytes can be activated to act as antigen presenting cells to promote anti-tumor responses | Rossetti et al. (11) | PLOS ONE | 2018 | muMT | TC-1 cell line | 15 |
| 12 | IL-33 delays metastatic peritoneal cancer progression inducing an allergic microenvironment | Perales-Puchalt et al. (12) | OncoImmunology | 2018 | muMT | ID8 cell line | 5 |
| 13 | Polynucleotides for inhibiting metastasis and tumor cell growth | Barbera-Guillem (13) | (US PATENT US6268350B1) |  | muMT | B16F10 | 2 |
| 14 | B cells Regulate Macrophage Phenotype and Response to Chemotherapy in Squamous Carcinomas | Affara et al. (14) | Cancer Cell | 2015 | JH−/− | SCC cell lines derived from K14-HPV16 mice | 198 |
|  |  |  |  |  |  |  |  |
| **Supporting** | **Title** | **Author (ref)** | **Journal** | **Year** | **Mouse model** | **Cancer type** | ***No. of Citations** |
| 1 | Anti-tumor immunity in B-lymphocyte-deprived mice. III. Immunity to primary Moloney sarcoma virus-induced tumors | Gordon et al. (15) | Int J Cancer | 1982 | anti-mouse IgM treatment | Moloney sarcoma virus (MSV) induced tumor | 23 |
| 2 | Low natural antibody and low in vivo tumor resistance, in xid-bearing B-cell deficient mice | Chow and Bennet (16) | The Journal of Immunology | 1989 | X-linked immunodeficiency-bearing B-cell deficient mice- low serum IgM and IgG3 | RI-28, a radiation-induced T cell leukemia | 25 |
| 3 | B Cells Are Required for Optimal CD4+ and CD8+ T Cell Tumor Immunity: Therapeutic B Cell Depletion Enhances B16 Melanoma Growth in Mice | DiLillo et al. (17) | J Immunol | 2010 | anti-CD20 mAb treatment | B16F1 | 263 |
| 4 | B cells promote tumor immunity against B16F10 melanoma | Kobayashi et al. (18) | Am J Pathol | 2014 | B-cell linker protein (BLNK) deficient (BLNK−/−) mice, in which B cell development and function is severely impaired | B16F10 | 29 |
| 5 | Immune Surveillance by Natural IgM Is Required for Early Neoantigen Recognition and Initiation of Adaptive Immunity | Atif et al. (19) | Am J Respir Cell Mol Biol | 2018 | IghelMD4 | B16F10  Urethane-induced tumors | 9 |
| 6 | Allogeneic IgG combined with dendritic cell stimuli induces anti-tumor T cell immunity | Carmi et al. (20) | Nature | 2015 | Anti-CD19 & anti-B220 mAb treatment | B16F10 | 134 |

**References**

1. Z. Qin *et al.*, B cells inhibit induction of T cell-dependent tumor immunity. *Nat Med* 4, 627-630 (1998).

2. A. I. Chapoval, J. A. Fuller, S. G. Kremlev, S. J. Kamdar, R. Evans, Combination chemotherapy and IL-15 administration induce permanent tumor regression in a mouse lung tumor model: NK and T cell-mediated effects antagonized by B cells. *J Immunol* 161, 6977-6984 (1998).

3. M. A. Perricone *et al.*, Enhanced efficacy of melanoma vaccines in the absence of B lymphocytes. *J Immunother* 27, 273-281 (2004).

4. S. Shah *et al.*, Increased rejection of primary tumors in mice lacking B cells: inhibition of anti-tumor CTL and TH1 cytokine responses by B cells. *Int J Cancer* 117, 574-586 (2005).

5. S. Inoue, W. W. Leitner, B. Golding, D. Scott, Inhibitory effects of B cells on antitumor immunity. *Cancer Res* 66, 7741-7747 (2006).

6. T. Tadmor, Y. Zhang, H. M. Cho, E. R. Podack, J. D. Rosenblatt, The absence of B lymphocytes reduces the number and function of T-regulatory cells and enhances the anti-tumor response in a murine tumor model. *Cancer Immunol Immunother* 60, 609-619 (2011).

7. E. B. Guillem, J. W. Sampsel, Immune-promoted tumor cell invasion and metastasis. New considerations in cancer therapy. *Adv Exp Med Biol* 532, 153-173 (2003).

8. Y. Zhang *et al.*, B lymphocyte inhibition of anti-tumor response depends on expansion of Treg but is independent of B-cell IL-10 secretion. *Cancer Immunol Immunother* 62, 87-99 (2013).

9. K. Y. Eckhard R.Podack, Nozomi Yamazaki and Joseph D.Rosenblatt (2013) ALLOGENEIC CANCER CELL-BASED IMMUNOTHERAPY. (The University of Miami, Miami, FL (US)).

10. A. Tang *et al.*, B cells promote tumor progression in a mouse model of HPV-mediated cervical cancer. *Int J Cancer* 139, 1358-1371 (2016).

11. R. A. M. Rossetti *et al.*, B lymphocytes can be activated to act as antigen presenting cells to promote anti-tumor responses. *PLoS One* 13, e0199034 (2018).

12. A. Perales-Puchalt *et al.*, IL-33 delays metastatic peritoneal cancer progression inducing an allergic microenvironment. *Oncoimmunology* 8, e1515058 (2019).

13. Barbera-Guillem (2001) POLYNUCLEOTIDES FOR INHIBITING METASTASIS AND TUMOR CELL GROWTH. in [*https://patents.google.com/patent/US6268350*](https://patents.google.com/patent/US6268350) (BioCrystal Ltd., Westervile, OH (US)).

14. N. I. Affara *et al.*, B cells regulate macrophage phenotype and response to chemotherapy in squamous carcinomas. *Cancer Cell* 25, 809-821 (2014).

15. J. Gordon, H. T. Holden, S. Segal, M. Feldman, Anti-tumor immunity in B-lymphocyte-deprived mice. III. Immunity to primary Moloney sarcoma virus-induced tumors. *Int J Cancer* 29, 351-357 (1982).

16. D. A. Chow, R. D. Bennet, Low natural antibody and low in vivo tumor resistance, in xid-bearing B-cell deficient mice. *J Immunol* 142, 3702-3706 (1989).

17. D. J. DiLillo, K. Yanaba, T. F. Tedder, B cells are required for optimal CD4+ and CD8+ T cell tumor immunity: therapeutic B cell depletion enhances B16 melanoma growth in mice. *J Immunol* 184, 4006-4016 (2010).

18. T. Kobayashi *et al.*, B cells promote tumor immunity against B16F10 melanoma. *Am J Pathol* 184, 3120-3129 (2014).

19. S. M. Atif *et al.*, Immune Surveillance by Natural IgM Is Required for Early Neoantigen Recognition and Initiation of Adaptive Immunity. *Am J Respir Cell Mol Biol* 59, 580-591 (2018).

20. Y. Carmi *et al.*, Allogeneic IgG combined with dendritic cell stimuli induce antitumour T-cell immunity. *Nature* 521, 99-104 (2015).
